# Role of Ox-PAPCs in the Differentiation of Mesenchymal Stem Cells (MSCs) and Runx2 and PPARγ2 Expression in MSCs-Like of Osteoporotic Patients

**DOI:** 10.1371/journal.pone.0020363

**Published:** 2011-06-03

**Authors:** Maria Teresa Valenti, Ulisse Garbin, Andrea Pasini, Mirko Zanatta, Chiara Stranieri, Stefania Manfro, Chiara Zucal, Luca Dalle Carbonare

**Affiliations:** Department of Medicine, Clinica di Medicina Interna, Sezione D, University of Verona, Verona, Italy; Brigham & Women's Hospital - Harvard Medical School, United States of America

## Abstract

**Background:**

Mesenchymal stem cells (MSCs) can differentiate into osteoblasts and adipocytes and conditions causing bone loss may induce a switch from the osteoblast to adipocyte lineage. In addition, the expression of Runx2 and the PPARγ2 transcription factor genes is essential for cellular commitment to an osteogenic and adipogenic differentiation, respectively.

Modified lipoproteins derived from the oxidation of arachidonate-containing phospholipids (ox-PAPCs: POVPC, PGPC and PEIPC) are considered important factors in atherogenesis.

**Methodology:**

We investigated the effect of ox-PAPCs on osteogenesis and adipogenesis in human mesenchymal stem cells (hMSCs). In particular, we analyzed the transcription factor Runx2 and the PPARγ2 gene expression during osteogenic and adipogenic differentiation in absence and in presence of ox-PAPCs. We also analyzed gene expression level in a panel of osteoblastic and adipogenic differentiation markers.

In addition, as circulating blood cells can be used as a “sentinel” that responds to changes in the macro- or micro-environment, we analyzed the Runx2 and the PPARγ2 gene expression in MSCs-like and ox-PAPC levels in serum of osteoporotic patients (OPs). Finally, we examined the effects of sera obtained from OPs in hMSCs comparing the results with age-matched normal donors (NDs).

**Principal findings:**

Quantitative RT-PCR demonstrated that ox-PAPCs enhanced PPARγ2 and adipogenic gene expression and reduced Runx2 and osteoblast differentiation marker gene expression in differentiating hMSCs. In OPs, ox-PAPC levels and PPARγ2 expression were higher than in NDs, whereas Runx2 was lower than in ND circulant MSCs-like.

**Conclusions:**

Ox-PAPCs affect the osteogenic differentiation by promoting adipogenic differentiation and this effect may appear involved in bone loss in OPs.

## Introduction

Osteoporosis is a common age-related skeletal disease characterized by loss of bone mass which increases bone fragility. Bone remodelling plays a major role in the maintenance of the mechanical integrity of the skeleton and involves a coordinated process of bone formation (promoted by osteoblasts) and bone resorption (promoted by osteoclasts) [1].

Osteoblasts derive from mesenchymal stem cells (MSCs) that are multipotent progenitor cells with the capacity to differentiate into connective tissue cells [2]. Mesenchymal stem cells can differentiate into osteoblasts but also in adipocytes and there is an inverse relationship between the formation of adipocytes and osteoblasts [3], [4]. Conditions associated with bone deficiency such as ovariectomy, treatment with glucocorticoids and immobilization are correlated with an increase of adipocytes in bone marrow [5], [6]. One hypothesis explaining bone loss suggests an imbalance in the differentiation of osteoblast and adipocyte progenitors at the expense of the osteoblasts; this hypothesis have been confirmed by histomorphometric analyses that have shown adipocytic replacement of the bone marrow in patients with osteoporosis [7].

Previous studies have shown that atherosclerotic calcification and osteoporosis may coexist in the same patient [8]. Hyperlipidaemia is associated with progression of coronary calcification and reduces bone mineral density in mice [9]. *In vitro* and *in vivo* studies show that oxidized lipids promote mineralization of the vascular cells and reduce mineralization of bone cells, inhibiting differentiation of pre-osteoblasts [8].

Recent findings suggest that postmenopausal women with atherogenic lipid profile show an elevated risk of osteopenia with respect to those with normal lipid profiles [10]. From another point of view, modified lipoproteins, particularly oxidized phospholipids, such as ox-PAPCs were considered an important factor in atherogenesis. Previous studies demonstrated ox-PAPCs inhibit spontaneous osteoblastic differentiation and mineralization of calvarial pre-osteoblasts and bone marrow stromal cells [8], [11].

The expression of transcription factor genes is essential for cellular commitment to a specific differentiation lineage [12] and activation of phenotype-specific transcription factors, such as osteoblast-specific Runx2/Cbfa1 and adipocyte-specific PPARγ2, determines lineage commitment [13], [14].

Runx2, a master gene for osteoblast differentiation, is a member of the runt family of transcription factors and its expression is necessary to differentiate and to activate osteoblasts [15]. Peroxisome proliferator-activated receptor gamma (PPARγ), a member of the nuclear receptor family of transcription factors, is important in the control of adipocyte development and in the glucose and fatty acid metabolism [16] and its activation has a pivotal role in selection of adipogenesis over osteoblastogenesis [17].

It has been shown that PPARγ2 is able to convert stromal cells from an osteoblastic phenotype to differentiated adipocytes and it can suppress the expression of Runx2 and osteoblast specific genes [18]. In addition, MSCs obtained from control and osteoporotic women show differences in their capacity to differentiate into osteogenic and adipogenic lineage [19].

On the basis of these acknowledges, we postulated that the gene expression alteration of Runx2 and PPARγ2 may disrupt the balance between osteo and adipo progenitors in osteoporotic patients with respect to normal individuals and that ox-PAPCs may contribute to these alterations.

In our previous study, we demonstrated the possibility to obtain mesenchymal stem cells-like (MSCs-like) from peripheral blood (PB-MSCs-like) by two-step method that leads the depletion of hematopoietic component [20] and the selection of circulant progenitors.

In order to investigate gene expression alterations and the contribute of ox-PAPCs in the pathogenesis of osteoporosis, we analyzed, *in vitro*, the gene expression during osteogenic and adipogenic differentiation in mesenchymal stem cells in presence or absence of ox-PAPCs. In addition, we analyzed the mRNA expression of both Runx2 and PPARγ2 in circulant MSCs-like, the levels of ox-PAPCs in serum of osteoporotic patients (OPs) and normal donors (NDs) and the adipogenic and osteogenic gene expression in hMSCs cultured with sera obtained from OPs and NDs.

Our study showed that ox-PAPC exposure affects the osteogenic and adipogenic differentiation of hMSCs *in vitro*. Furthermore, ox-PAPC levels resulted higher in OPs with respect to NDs and the sera obtained from OPs affect the differentiation process in hMSc.

## Results

### Effects of ox-PAPC treatment in mesenchymal stem cells

**Figure 1:**
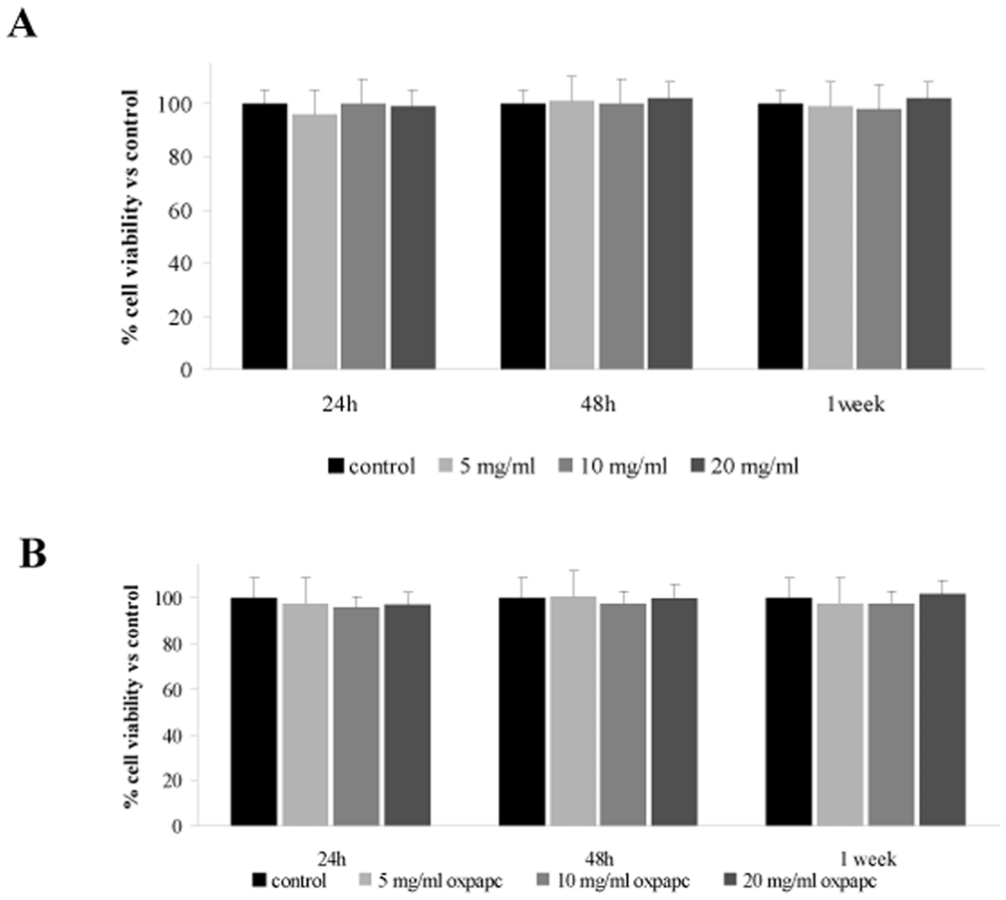
Cell viability in hMSCs, expressed in percentage. XTT test was performed in hMSCs treated with and without ox-PAPCs during adipogenic (A) and osteogenic (B) differentiation (n = 3).

To assess the effect of the ox-PAPC treatment, mesenchymal stem cells with specific differentiation medium in absence and in presence of ox-PAPCs ranging from 5 to 20 µg/ml were evaluated. Cell viability as measured by the XTT assay was similar during adipogenic and osteogenic differentiation with and without ox-PAPC treatment (Figure 1).

When gene expression analysis was performed, we observed that the treatment with 10 µg/ml up to 24 h during adipogenic differentiation had no effect on *PPARγ2* gene expression, whereas after 48 h the treatment induced an upregulation of this gene with respect to untreated cells (Table 1A) suggesting that further modification, internalization, or processing of ox-PAPCs may be necessary to obtain this effect. The adipogenic differentiation specific gene (*AdipoQ and Lep*) resulted up-regulated with respect to the untreated cells only after 1 week of ox-PAPC treatment (Table 1B and Table S1).

**Table 1:** Fold change mRNA levels in hMSCs treated with ox-PAPCs during adipogenic differentiation.

A			
(*PPARγ2*)	Ox-PAPCs 5 µg/ml	Ox-PAPCs 10 µg/ml	Ox-PAPCs 20 µg/ml
**24 h treatment**	1.03	1.02	1.10
**48 h treatment**	1.05	1.38*	1.37*

B			
1 week			
***PPARγ2***	1.18	1.42*	1.47*
***AdipoQ***	1	1.24*	1.25*
***Lep***	1.08	1.28*	1.3*

*p<0.01.

**Figure 2:**
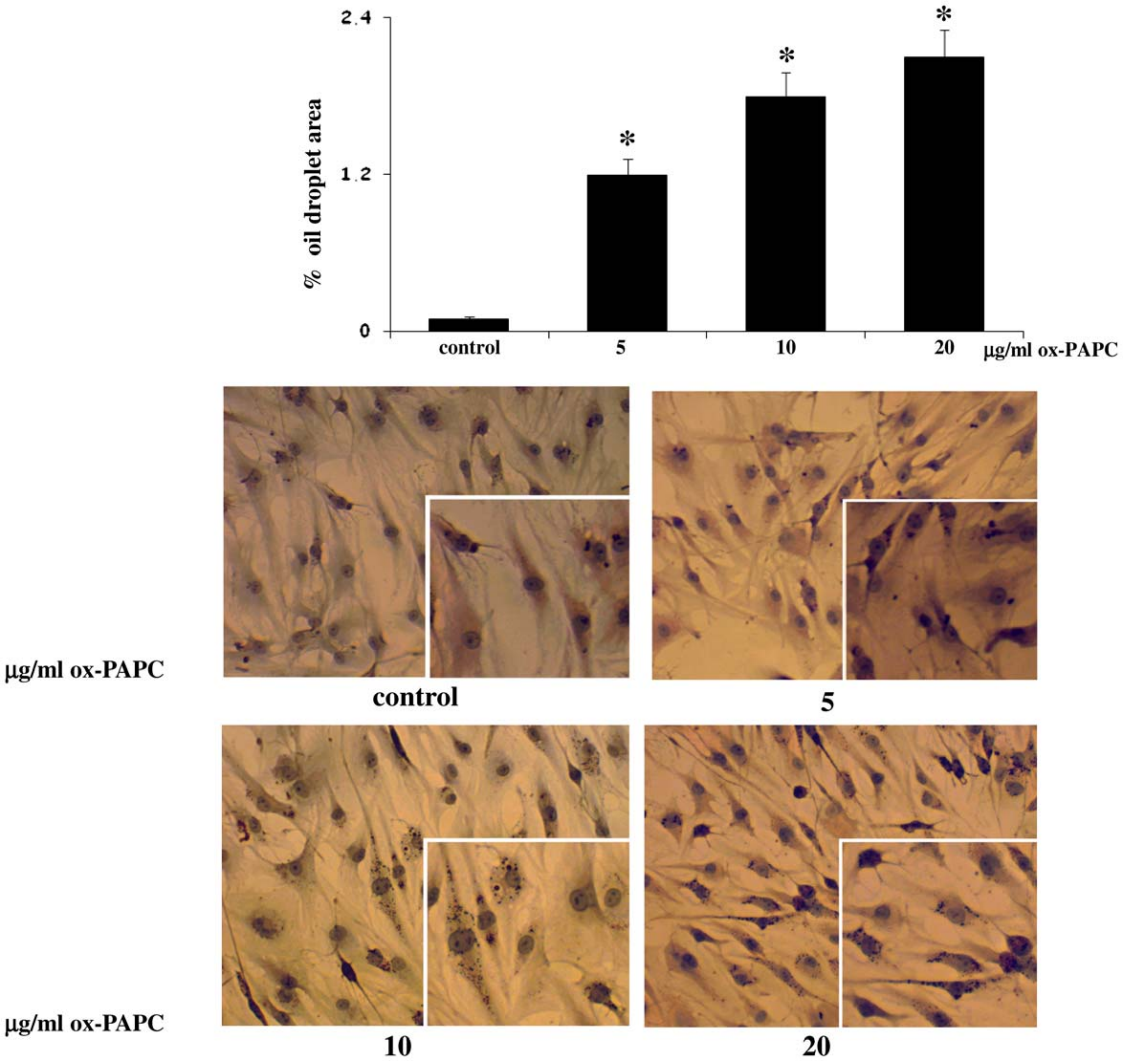
OIL Red O staining. When the hMSCs were cultured for 72 h in presence of ox-PAPCs during adipogenic differentiation, they showed a dose dependent significant increase of lipid-folled droplets (p<0.05). (original magnification 20×, insert 40×). The percentage of area stained with OIL Red O (red droplets) was quantified by using the IMAGE J Software.

In addition, when cells were cultured for 72 h in presence of ox-PAPCs during adipogenic differentiation, OIL Red O staining showed an increase of intracellular lipid-filled droplets in a dose dependent manner (Figure 2). In particular, the area of oil red O staining was 0.1%±0.05 in the control and 1.2%±0.6, 1.8%±0.5 and 2.1%±0.5 at 5 µg/ml, 10 µg/ml and 20 µg/ml, respectively.

After 1 week of ox-PAPC treatment, the percentage of area stained by oil red O was: 18%±2 in control, and 25%±2.7, 34%±1.6 and 42%±3.6 at 5 µg/ml, 10 µg/ml and 20 µg/ml, respectively.

On the contrary, during osteogenic differentiation the ox-PAPC treatment induced a downregulation on *Runx2* gene expression from 24 h and this effect was present also after 48 h of treatment (Table 2A). In particular, gene expression of *Runx2* during osteogenic differentiation was downregulated in a dose dependent manner with ox-PAPC treatment for 48 h at a concentration ranging from 5 to 20 µg/ml (Table 2A).

**Table 2:** Fold change mRNA levels in hMSCs treated with ox-PAPCs during osteogenic differentiation.

A			
(*Runx2*)	Ox-PAPCs 5 µg/ml	Ox-PAPCs 10 µg/ml	Ox-PAPCs 20 µg/ml
**24 h treatment**	1.04	0.64*	0.65*
**48 h treatment**	0.94	0.56*	0.45**

B			
1 week	Ox-PAPCs 5 µg/ml	Ox-PAPCs 10 µg/ml	Ox-PAPCs 20 µg/ml
***Runx2***	0.87	0.42**	0.44**
***Colia1***	0.82	0.54*	0.42**
***Sparc***	0.85	0.54*	0.42**
***Spp1***	0.87	0.5*	0.44**

*p<0.01;

**p<0.005.

**Figure 3:**
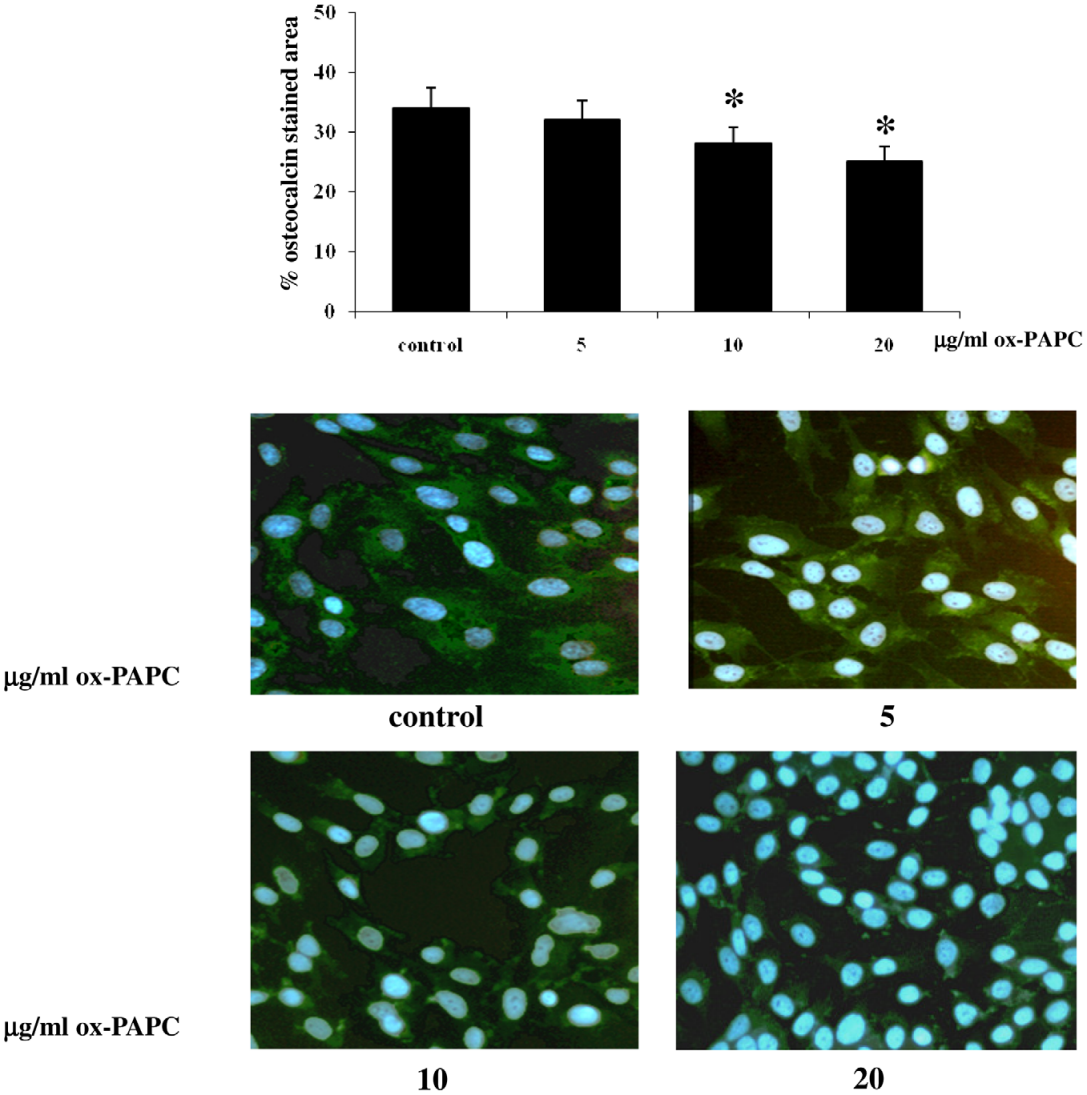
Osteocalcin immunofluorescence. After 10 days of ox-PAPC treatment during osteogenic differentiation, osteocalcin expression decreased in hMSCs in a dose dependent manner (n = 3; p<0.05). (original magnification 20×).

After 48 h and 1 week of osteogenic differentiation in absence or in presence ox-PAPCs in hMSCs, we performed the expression analyses for bone specific genes. According to Runx2 levels at the beginning of osteogenic commitment, we observed a downregulation of collagen type 1 (*Colia1*), osteonectin (*Sparc*) and osteopontin (*Spp*1) gene expression in hMSCs cultured with osteogenic differentiation medium in presence of ox-PAPCs at a concentration ranging from 5 to 20 µg/ml only after 1 week (Table 2B and Table S1). When we evaluated the osteocalcin expression by immunofluorescence, we observed a significant protein level reduction 10 days after ox-PAPC treatment (Figure 3).

To evaluate the effects of oxPAPCs in undifferentiated cells, we treated hMSCs cultured in medium w/o adipogenic and osteogenic differentiation factors with ox-PAPCs at a concentration ranging from 0 (controls) to 20 µg/ml.

When we performed the expression analysis for adipogenic and osteogenic genes, we observed a significant increase of PPARγ2 gene after 1 week of treatment (Table 3C). All other genes tested didn't change the value of expression with respect to untreated controls (Table 3A and 3B). The level of AdipoQ and Lep genes were less expressed in all conditions tested.

**Table 3:** Fold change mRNA levels in undifferentiated hMSCs treated with ox-PAPCs.

A			
24 h treatment	Ox-PAPCs 5 µg/ml	Ox-PAPCs 10 µg/ml	Ox-PAPCs 20 µg/ml
***PPARγ2***	1.05	1.01	1.12
***Runx2***	1.05	0.98	1.01
***Spp1***	1.05	0.95	1.01
***Colia1***	0.98	0.97	0.97
***Sparc***	1.01	0.98	1.02

B48 h treatment			
***PPARγ2***	1.06	1.16	1.18
***Runx2***	0.97	1.02	1.01
***Spp1***	1.03	0.98	1.02
***Colia1***	1.01	1.02	0.96
***Sparc***	1.01	1.04	1.02

C			
1 week treatment			
***PPARγ2***	1.08	1.18	1.31*
***Runx2***	1.01	0.98	0.97
***Spp1***	1.05	0.97	1.01
***Colia1***	0.98	0.97	1.02
***Sparc***	1.02	1.01	0.98

*p<0.05.

### Densitometric results in OPs and NDs and circulant progenitor cell phenotype

Densitometric results are showed in Table 4. OP patients showed bone densitometric values lower than normal donors at all skeletal sites evaluated.

**Table 4:** Densitometric and biochemical data of the study population.

Variables	OP	ND	p
Number	34	25	
Age (years)	59.12±5,61	58,39±8,20	ns
BMD spine (g/cm^2^)	0,732±0,04	0,958±0,14	*
T sc lumbar spine (SD)	−2,87±0,36	−1.00±1,07	*
Z sc lumbar spine (SD)	−1,52±0,53	0,21±1,26	*
BMD femoral neck (g/cm^2^)	0,64±0,06	0,735±0,09	*
T sc femoral neck (SD)	−1,79±0,67	−1,07±0,63	*
Z sc femoral neck (SD)	−0,56±0,77	0,02±0,7	*
BMD total hip (g/cm^2^)	0,75±0,06	0,853±0,05	*
T sc total hip (SD)	−1,51±0,65	−0,81±0,54	*
Z sc total hip (SD)	−0,63±0,70	0,07±0,65	*
Cholesterol (mg/dl)	203±58	212±44	ns
C Reactive Protein (CRP, mg/L)	<3	<3	ns

Values are expressed as mean ± SD.

*p<0.001 between PO and ND.

The isolated circulant progenitor cells from osteoporotic patients and normal donors expressed the cluster differentiation markers in a similar percentage. In particular, the selected cells were CD3^−^, CD19^−^, CD34^low^; the CD14^+^ resulted 0.5% (±0.07) in osteoporotic patients vs 0.47% (±0.08) (p = NS), CD45^+^ 2.25% (±0.3) vs 2.29 (±0.45) (p = NS). In addition, the mRNA related to osteoblast (*Colia1 and Spp1*) and adipocyte (*AdipoQ, and Lep*) specific markers were not detectable in circulant MSCs-like. In fact, as previously reported (20), these circulant MSCs-like obtained by depletion method don't contain well differentiated osteoblastic cells. In addition, cells were negative also for *AdipoQ* and *Lep* expression. However, these cells were positive for osteogenic and adipogenic transcription factor genes (Runx2 and PPARγ, respectively) suggesting an early phase of their commitment.

### Transcription factor gene expression in circulant MSCs-like and level of ox-PAPCs in peripheral blood

**Figure 4:**
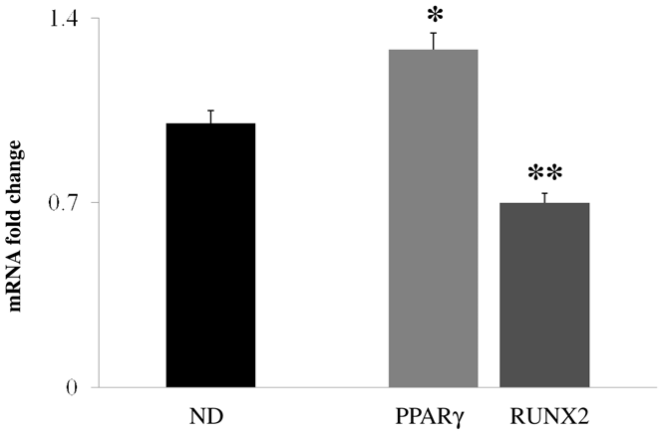
mRNA fold change. PPARγ2 and Runx2 in circulant MSCs-like from normal donors (NDs) and osteoporotic patients (OPs), * p<0.005 vs NDs and **p<0.001 vs NDs, respectively. The fold change was obtained by using three different housekeeping genes (GAPDH, β2 microglobulin, beta-actin).

Results of this study revealed that *PPARγ2* and *Runx2* mRNA have a different expression in MSCs-like of osteoporotic patients with respect to aged matched normal donors. All patients and normal donors were females and no smokers. Peripheral blood was collected and MSCs-like were isolated by depletion method for the total RNA extraction and for reverse transcription in cDNA. Figure 4 shows the expression analysis of the *PPARγ2* and *Runx2* genes by the real-time PCR. The difference fold change of expression of *PPARγ2* and *Runx2* mRNA (normalized by using three different housekeeping genes, GAPDH, β2 microglobulin, and beta-actin genes, respectively) in PB-MSCs-like of OPs compared to NDs were 1.28±0.14 and 0.7±0.07, respectively. The expression of *PPARγ2* mRNA in PB-MSCs-like of OPs was significantly higher than NDs (p<0.005), whereas the *Runx2* expression in osteoporotic patients resulted lower than in normal donors (p<0.001).

**Figure 5:**
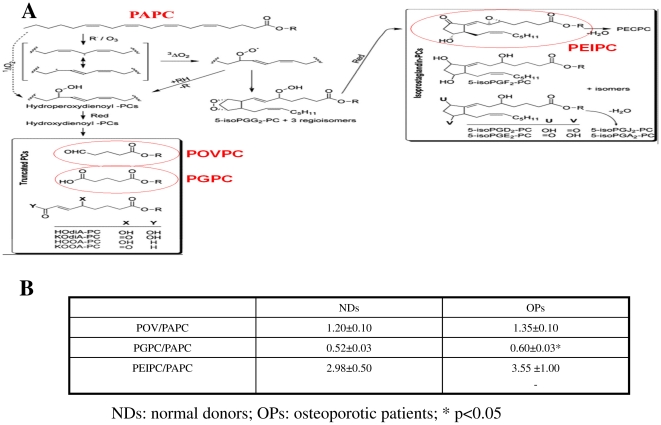
Ox-PAPCs in peripheral blood. A) Representation of autoxidised reaction of 1-palmitoyl-2-arachidonoyl-sn-glycero-3-phosphocholine. B) Levels of POVPC, PGPC and PEIPC (ox-PAPCs) in sera of normal donors (NDs) and osteoporotic patients (OPs). PGPC form resulted significantly higher in osteoporotic patients with respect to normal donors (p<0.05).

All three chemical compounds (POV/PAPC, PG/PAPC and PEIP/PAPC) were detectable in serum of osteoporotic patients (1.35 ng/mg, 0.6 ng/mg, 3.55 ng/mg, respectively) and in normal donors (1.2 ng/mg, 0.52 ng/mg, 2.98 ng/mg, respectively), even if only PG/PAPC resulted significantly higher in OPs than in NDs (p<0.05) (Fig. 5 and Table S2).

### Effects of sera on osteogenic and adipogenic differentiation

To evaluate the effects of sera obtained from OPs or NDs during differentiation in MSCs, we used randomly pooled sera either from OPs and NDs, as described in material and method sections. Under these conditions, cells were cultured for 24 h, 48 h and 1 week before cell viability and gene expression analysis evaluation.

Under all conditions assayed, no viability differences were observed with respect to pooled sera from OPs and from NDs (data not shown).

When gene expression was analyzed, until 24 h of sera treatment the expression levels for all genes tested were similar in all cases (Table 5). After 48 h of incubation with sera from OPs or NDs in cells cultured in adipogenic medium, levels of expression of *PPAR*γ*2* gene were similar (Table 5).

**Table 5:** Fold change mRNA levels in hMSCs treated with OP vs. ND pooled sera during adipogenic (*PPARγ2*, *ADIPOQ* and *LEP*) and osteogenic (*RUNX2*, *SPP1*, *COLIA1*, *SPARC*) differentiation.

	24 h treatment	48 h treatment	1 week treatment
***PPARγ2***	1.03	1.11	1.3*
***AdipoQ***	1.01	1.02	1.18
***Lep***	1.01	1.02	1.12
***Runx2***	0.98	0.92	0.92
***Colia1***	0.97	0.84	0.83
***Sparc***	1.01	0.80*	0.78*
***Spp1***	1.05	0.76*	0.72*

*p<0.05.

However, after 1 week of cell culture, the expression of PPARγ2 resulted higher in hMSCs with respect to NDs sera in presence of OP sera (Table 5).

During osteogenic differentiation, the effects of OP sera in osteogenic gene expression were more relevant (Table 5) than in adipogenic gene expression. Values of each serum pool are reported in Table S3.

## Discussion

To analyze the effect of ox-PAPCs in the osteogenic and adipogenic differentiation, we used an *in vitro* model based on commercial MSCs to eliminate the potential effect of different circulant growth factors and cytokines.

Our data showed that the ox-PAPC treatment affects the osteogenic and adipogenic differentiation by altering the transcription factor and the lineage related gene expression in a dose dependent manner and by promoting the adipogenic differentiation at the expense of osteogenic differentiation. Even if fold variations of the specific gene expressions were significant, differences were small and this aspect could limit the biological relevance of the results. Nevertheless, a different level of Oil red O stained areas and a number of positive osteocalcin cells between control and treated samples are consistent with observed differences in gene expression, thus supporting a biological role of the ox-PAPCs. Indeed, ox-PAPC effects on gene expression promoted an adipogenic phenotype with increased intracellular lipid-filled droplets and lessened the osteogenic phenotype, as confirmed by decreased osteocalcin levels.

It has been reported that the adipogenic differentiation depends on PPARγ2 expression associated with the availability of PPARγ ligand/activator [21]. The same authors showed that oxidized derivatives of linoleic acid present in oxidized LDL promoted adipogenic differentiation by activating PPARγ in an *in vitro* model [22] suggesting ox-PAPCs as potential activators of PPARγ. In addition, another study *in vitro* focused on the research of potential PPARγ activators showed that ox-PAPCs and its components POVPC and PGPC were able to activate PPAR response element-driven report. Ox-PAPCs might move to the nucleus for binding PPAR receptor or might activate PPAR receptor by its hydrolytic products [23].

It is known the ox-PAPCs may have opposite effects on different cells. Bear and coworkers showed that ox-LDL may promote osteogenic differentiation in vascular smooth muscle cells with consequent vascular calcification *in vitro* [24]. On the contrary, in a previous study, in addition to effects of lipid oxidation products on calcifying vascular cells, it was shown that treatment of preosteoblastic MC3T3-E1 cells with MM-LDL inhibited their differentiation, as indicated by inhibition of alkaline phosphatase activity and calcification [8]. These evidences support epidemiological data suggesting a possible link between cardiovascular diseases associated to lipidic alterations and osteoporosis. However, the relationship between skeleton and cardiovascular system is certainly complex and involved several aspects, as suggested by recent findings [25], [26] and by the fact that not all osteoporotic patients have lipid disorders and vice versa.

The interactions between peripheral blood cells and the entire body lead the possibility that changes occurring in association with the diseases, within the body tissues, may trigger specific alterations in gene expression in blood cells reflective of the stimulus. A recent study suggested that circulating blood cells can be used as a “sentinel” that responds to changes in the macro- or micro-environment in organs, making the blood an ideal surrogate tissue for diagnostic analyses [27].

Our previous study demonstrated that circulating MSCs-like showed an abnormal osteogenic differentiation in osteoporotic patients [28]. In effect, we observed a downregulation of the master gene Runx2 and the downstream factor Osterix and also the mRNA of *Sparc*, *Spp1* and *Colia1* resulted lower in osteoporotic patients with respect to age-matched normal donors. In addition, we observed a significant correlation between bone density and osteoblastic gene expression, suggesting a direct relationship between bone mass and osteoblast differentiation and confirming an involvement of osteoblastic lineage in the mechanism of bone loss [28].

Osteoblasts and adipocytes have a common mesenchymal progenitor and an inverse relation between the two lineages under some pathological conditions has already been suggested [29]. Adipocyte differentiation is controlled by peroxisome proliferators-activated receptors (PPARs), members of the nuclear receptor superfamily [30] and analyses of home- and heterozygous PPARγ-deficient mice have suggested that PPARγ may determine the fate of osteo-adipocyte precursor during differentiation events [31]. *Runx2* (also known as *Cbfa1*) is known to be essential for osteoblastic differentiation, because its null mutation in mice exhibits the complete lack of bone [32]. *Runx2* expression is upregulated in proliferative chondrocytes and it is early expressed in osteoblastic differentiation. This gene can be stimulated by multiple signal transduction pathways [33] and it can directly stimulate the transcription of osteoblast-related genes [34].

Mesenchymal stem cells obtained from bone marrow of donors and osteoporotic patients showed differences in their capacity to differentiate into the osteogenic and adipogenic lineage [35] and, considering that osteoblastic and adipogenic differentiation pathway are regulated jointly, this observation suggests that marrow adipogenesis could be involved in some osteogenic disorders [30], [36].

On the basis of these findings, we hypothesized that adipogenic and osteogenic transcription factor gene expression in mesenchymal precursors may be different in osteoporotic patients with respect to normal donors. According to this suggestion, we observed a different expression profile of transcription factor genes (*PPARγ2* and *Runx2*) in circulant MSCs-like of osteoporotic patients with respect to normal donors. Indeed, the overexpression of *PPARγ2* mRNA was associated to a downregulation of *Runx2* mRNA in osteoporotic patients with respect to normal donors. It is known that in age-related and postmenopausal osteoporosis, adipogenic differentiation of progenitor cells increases at the expense of osteogenic differentiation [37] and our results showed that the different levels of *Runx2* and *PPARγ2* gene expression in MSCs-like may reflect this shift. Although the causes inducing this shift are not yet clearly identified, the fate of progenitors cells may be determined by paracrine and autocrine factors acting on the control of differentiation master regulators. In addition, difference in the composition of fatty acid may contribute to this shift since it has been demonstrated that specific fatty acids are able to activate the adipogenesis master regulator PPARγ [3]. This is in agreement with our results as both the gene expression of *PPARγ2* and the levels of ox-PAPCs are higher in osteoporotic patients with respect to normal donors.

It has been shown that PPARγ2, upon activation by ligand binding, regulates the transcription of adipocyte-specific genes [38] and its activation by free fatty acid, prostaglandin J2 and the thiazolidinedione class of antidiabetic drugs induces adipogenesis [3].

Products of lipid and lipoprotein oxidation including minimally oxidized low density lipoprotein (MM-LDL) inhibit the differentiation and mineralization of preosteoblasts *in vitro* [8].

The results of the present study, showed that all the three ox-PAPC forms, (POV/PAPC, PG/PAPC and PEIP/PAPC) are detectable in serum either in osteoporotic patients either in normal donors. However, only PG/PAPC resulted significantly higher in osteoporotic patients than in normal donors.

It is known that atherogenic lipoproteins and phospholipids, including midly oxidized LDL and ox-PAPCs, inhibit osteogenesis in bone marrow stromal cells via the ERK pathway [11]. According to these findings, in addition to an increase of ox-PAPCs in serum of osteoporotic patients, our data showed that the master gene of osteogenic differentiation Runx2 was significantly lower in their circulant MSCs-like. Moreover, the serum obtained from OP patients, affects the differentiation process by altering the transcription factor *Runx2* and *PPAR*γ*2* gene expression. Even if we cannot exclude the role of various cytokines circulating in peripheral blood, nevertheless we hypothesize that ox-PAPCs could be involved in osteogenic differentiation.

### Conclusions

The alterations in transcription factor gene expression during differentiation triggered by ox-PAPCs support the hypothesis that modified lipoproteins are involved in inhibition of early osteogenic differentiation from mesenchymal stem cells. This inhibition can promote adipogenic cell population and this aspect can support the hypotesis that lipid alteration is associated with osteoporosis. In addition, data *in vivo* showing a different transcription gene expression pathway in association with a higher ox-PAPC levels in osteoporotic patients with respect to normal donors suggest that oxidation lipid products may contribute, at least in part, to the fate of progenitor cells with a consequent bone loss. In fact, it is known that oxidized lipids accumulate within the skeleton and, in particular, within the perivascular space of Haversian canals in osteoporotic patients [39]. However, further studies should be performed in order to evaluate the relationship between osteogenic and adipogenic pathways.

## Materials and Methods

### Ethics statement

Written informed consent was obtained from all participants and the study was approved by the Ethical Committee of Azienda Ospedaliera Universitaria Integrata of Verona, Italy.

### Patients

We selected 34 postmenopausal osteoporotic patients (OPs) consequently referred to our Metabolic Bone Disease Centre from January 2006 until November 2008. We compared these patients with 25 normal postmenopausal women (NDs) matched for age and anthropometric parameters. None of patients and volunteers was taking any therapy before the enrollement.

All women included in the study underwent a densitometric (DXA) measurement at the lumbar spine and hip (Hologic QDR Discovery Acclaim, Waltham, Massachusetts, USA) and osteoporosis was diagnosed according to WHO parameters, considering as the cut-off that a lumbar spine or femoral T score lower than −2.5 SD [40].

Biochemical evaluations were performed in all osteoporotic patients in order to rule out secondary causes of osteoporosis (S-calcium [Ca], S-phosphate [P], urinary calcium rate [CaE], Parathyroid Hormone [PTH], carboxy-terminal telopeptide of type I collagen [CTX], alkaline phosphatase [ALP], 25-hydroxyvitamin D [VitD]).

Normal donors inclusion criteria were a T sc>−2 SD without any history of bone fractures. In addition, in both osteoporotic patients and normal donors groups, cholesterol levels and CRP (C Reactive Protein) were evaluated.

Women with history of premature menopause (<45 year old), traumatic fractures in the previous 12 months or with diseases or using drugs able to affect bone or calcium-phosphate metabolism were excluded from the study.

### Cell culture

Human mesenchymal stem cells obtained from PromoCell (C-12974 International pbi, Milano, Italia) derived from normal human bone marrow and are tested for their ability to differentiate *in vitro* into adipocytes, chondrocytes and osteoblasts (as per Promocell).

We used commercial MSCs to have a sufficient quantity of cells to perform all experiments and to eliminate the potential effect of different circulant growth factors and cytokines.

Cells were cultured in Mesenchymal Stem Cell Growth Medium containing Basal Medium and SupplementMix (from PromCell) and supplemented with 100 U/ml penicillin and 100 U/ml streptomycin (from Invitrogen).

For use in the experiments the hMSCs were plated at a density of 5×10^4^ cells per well into 48-well plates or 5×10^3^ cells per well into 96-well plates. After two days, the medium was removed and osteogenic or adipogenic differentiation was induced with Mesenchymal Stem Cell Osteogenic Differentiation Medium or Stem Cell Adipogenic Differentiation Medium (all from PromoCell).

### Ox-PAPC treatment

PAPC**s** was oxidized by transferring 1 mg in 100 µl of chloroform to a clean glass test tube and evaporating the solvent under a stream of nitrogen. The lipid residue was allowed to autoxidize while exposed to air for 72 h at room temperature. The extent of oxidation was monitored by flow injection ESI-MS. After oxidation the medium used in the experiments was added.

The cells were cultured with specific differentiation medium in absence and in presence of ox-PAPCs ranging from 5 to 20 µg/ml. After the treatment, the cells were used for analyses.

### Treatment of hMSCs with human sera

We used the hMSCs purchased from PromoCell in order to analyze the effects of sera obtained from OPs and from NDs during adipogenic and osteogenic differentiation. We used commercial MSCs to eliminate the potential effect of different circulant growth factors and cytokines.

In particular, we randomized sera from OPs and NDs, as reported in previous studies [41]–[44]. Briefly, serum samples from 34 OPs were divided into six groups. In the same way, serum samples from 25 NDs were randomly divided into five groups. Each serum pool was obtained with equal volumes from all patients or all donors within the group.

We added the pooled sera to osteogenic and adipogenic medium at 10% concentration. Cells were plated at a density of 5×10^4^ cells per well into 48-well plates and cultured for 24 h, 48 h and 1 week before gene expression analysis.

### Cell viability

Cell viability was evaluated by a colorimetric assay based on the reduction of the tetrazolium salt XTT (sodium 3I-[1-phenylamino- carbonyl-3,4-tetrazolium]-bis(4-methoxy-6-nitro)benzene sulfonic acid hydrate) by mitochondrial dehydrogenase of viable cells to a formazan dye (Cell proliferation kit II — XTT Roche). Briefly, 5×10^3^ cell/well in 96 microtitre plates were treated, once 80% confluence was reached, at concentrations ranging from 5 to 20 µg/ml of ox-PAPCs for 24 h, 48 h and 1 week. After the incubation period, 100 µl XTT labelling mixture was added to each well and incubated at 37°C under humidified atmosphere of 5% CO_2_ for 4 h. The spectrophotometric absorbance of the samples was measured using a microtitre plate (ELISA) reader at a wave length of 450 nm.

### Oil red O staining

The cells, cultured with adipogenic medium in absence or in presence of ox-PAPCs ranging from 5 to 20 µg/ml, were stained with Oil Red O and the total area of red pixels in the Oil Red O-stained droplets/cell was determined by using the IMAGE J image analysis program developed at the National Institutes of Health. In particular, the mean ± SD of red stained area, measured at magnification of 40× in six cell-containing slides and three different field/slide from each group, were expressed as percentage respect to total area.

### Immunofluorescence

For evaluation of osteocalcin, cells were grown in slide with osteogenic medium in absence or in presence of ox-PAPCs ranging from 5 to 20 µg/ml for 10 days under humidified atmosphere of 5% CO_2_ at 37°C; then, cultured cells were fixed for 20 min in 2% paraformaldehyde and stored a −20°C.

The slides were washed three times in PBS and permeabilized with 0.2% Triton X-100 in PBS. After three washs in PBS, cells were blocked for 1 h in blocking buffer containing 1% of bovine serum albumin (BSA) in PBS. Cells were washed again and incubated overnight with the primary antibody (osteocalcin mouse monoclonal antibody, Santa Cruz Biotechnology, Inc Switzerland). After three washes in PBS, cells were incubated for 2 h in fluoroisothiocyanate (FITC) conjugated secondary antibody (goat antimouse igG, Santa Cruz Biotechnology, Inc Switzerland). Cells were washed again three times with PBS and counterstained with 4,6 diamidino-2-phenylindole (DAPI) to detect nuclei and mounted with aqueus mounting medium.

To express in a semiquantitative way the levels of protein, the total area of green pixels (corresponding to osteocalcin positive area) was determined by using the IMAGE J image analysis program developed at the National Institutes of Health as above described.

### Sample collection for serum and circulant MSCs-like

A sample of peripheral blood was obtained by venipuncture from each of 34 OPs and 25 NDs. For each OP and ND serum and cells were obtained. The serum was obtained from 10 ml of fresh blood, obtained by ven**i**puncture, by centrifugation at 400×g. the serum was harvested and separated into aliquots and frozen at −80°C until the use. MSC-like cells were isolated by starting from 50 ml using a method including two ficoll procedures to deplete hematopoietic cells by antibodies cocktail. Firstly, mononuclear cells from 50 ml of heparinizated peripheral blood by gradient centrifugation (Ficoll GE) were obtained. The enriched cell pellet (concentrated mononuclear cells) was mixed with 4 ml of additional peripheral blood (from the same patient) as red cells are necessary to apply 200 µl antibodies cocktail (RosetteSep Mesenchymal Enrichment Cocktail; StemCells); this mix contains bi-specific antibody complexes against red blood cells (glycophorin A), CD3, CD14, CD19, CD38 and CD66b positive cells (Stem Cell Technologies Inc). These antibody complexes crosslinked the unwanted cells with red blood cells in the sample, causing them to pellet together when centrifuged over the second ficoll.

The enriched cells obtained were washed in PBS and analyzed for transcripts expression.

### Analysis of cell phenotype

We analyzed the cell phenotype at the RNA level for CD3, CD14, CD19, CD45, and CD34 markers, as previously described [45]. This method allows the phenotypic analysis in small number of cells obtained by stringent stem-cell purification strategies [45]. In addition, we evaluated in circulant MSCs-like also the mRNA expression of osteoblast (Colia1, and Spp1) and adipocyte (AdipoQ and Lep) markers. RNA extraction and RT Real-Time PCR were performed in circulant MSCs-like samples from osteoporotic patients and normal donors as following described.

### Total RNA extraction

Total RNA was extracted from each cell pellet from MSCs-like of OPs and NDs and from *in vitro* cell cultures by using the RNAeasy minikit (Quiagen) with DNAse I treatment. The amount of extracted RNA was quantified by measuring the absorbance at 260 nm. The purity of the RNA was checked by measuring the ratio of the absorbance at 260 and 280 nm, where a ratio ranging from 1.8 to 2.0 was taken to be pure. The absence of degradation of the RNA was confirmed by RNA electrophoresis on a 1.5% agarose gel containing ethidium bromide.

### Reverse transcription

First-strand cDNA was generated using the First Strand cDNA Synthesis Kit (GE Healthcare), with random hexamers, (GE Healthcare) according to the manufacturer's protocol. RT product was aliquoted in equal volumes and stored at −80°C.

### Real time RT-PCR

PCRs were performed in a total volume of 50 µl containing 1× Taqman Universal PCR Master mix, no AmpErase UNG and 5 µl of cDNA from each sample; pre-designed, Gene-specific primers and probe sets for each gene (CD3, Hs00174158_m1; CD14, Hs02621496-s1; CD19, Hs00174333_m1; CD45, Hs00174541_m1; CD34, HS00156373_m1; *Runx2*, Hs00231692_m1; *PPARΓ*, Hs01115513_m1; B2M, Hs999999_m1; COLLAGEN, TYPE I, ALPHA 1 (*Colia1*) Hs00164004_m1; OSTEONECTIN (*Sparc*) Hs00234160_m1; OSTEOPONTIN (*Spp1*) Hs00167093_m1; ADIPONECTIN (*AdipoQ*) Hs00605917_m1; LEPTIN (*Lep*) Hs00174877_m1;, GAPDH, 0802021; beta-actin, 0807024; Applied Biosystems) were obtained from Assay-on-Demande Gene Expression Products (Applied Biosystems). Real Time RT-PCR reactions were carried out in two-tube system and in multiplex. The Real Time amplifications included 10 minutes at 95°C (AmpliTaq Gold activation), followed by 40 cycles at 95°C for 15 seconds and at 60°C for 1 minute. Thermocycling and signal detection were performed with ABI Prism 7300 Sequence Detector; (Applied Biosystems). Signals were detected according to the manufacturer's instructions. This technique allows the identification of the cycling point where PCR product is detectable by means of fluorescence emission (Threshold cycle or Ct value). As previously reported, the Ct value correlates to the starting quantity of target mRNA [46]; we selected the ΔRn in the exponential phase of amplification plots to determine the Ct values and to obtain the linearity of calibration curves.

To evaluate the cell phenotype, mRNA quantification was analyzed by Relative Standard Curve Method. The amount of cDNA in each sample, obtained as average of triplicates and normalized with beta-2-microglobulin (B2M), was determined from standard curve derived from a four cDNA dilution series (from peripheral blood cells) and the results are expressed as percentage for expression of cluster differentiation marker genes. The criteria for evaluate the presence of mRNA related to osteoblast and adipocyte markers are based on the mRNA detectability in circulant MSCs-like by using the osteogenic and adipogenic differentiated MSC line as a control.

To compare relative mRNA levels in the OP and ND and *in vitro* treated cells, the gene expression was calculated after normalization against the housekeeping genes (B2M, GAPDH and β-actin) using the relative fold expression differences [47].

Ct values for each reaction were determined using TaqMan SDS analysis software. For each amount of RNA tested triplicate Ct values were averaged. Because Ct values vary linearly with the logarithm of the amount of RNA, this average represents a geometric mean. In addition, we considered as relevant a fold change <0.8 and >1.2, even if also smaller changes were statistically significant.

### Phospholipid extraction

A total lipids fraction was obtained after liquid-liquid extraction of 120 µl specimen of blood serum with chloroform-methanol mixture (3∶1, v/v), according to the method of Folch [48]. Blood serum was shaken for 10 min at room temperature, then, 0.9% sodium chloride solution was added there to, the extract was shaken for 30 s and immediately centrifuged (1500×g/5 min/4°C). The lower layer of the lipid extract was separated and dried under N2, and then dissolved in 40 µl of a chloroform-methanol mixture (1∶4 v/v, 0.1% BHT).

### Analysis of oxidized phospholipid levels

We analyzed the three ions, m/z 594.3 POVPC, 610.2 PGPC and 828.5 PEIPC as NP-HPLC fraction that contained these species possessed biological activity in peripheral blood [49]. Samples in 40 µl of chloroform-methanol mixture were injected onto a C8 column (4,6×250 mm, particle size 5 µm, Waters Spherisorb) equilibrated in 95% MeOH containing 1 mM ammonium acetate. The column was eluted isocratically at 0.3 ml/min. Mass-spectrometry was performed using an Agilent 1100 Ion Trap mass analyzer, fitted with an ESI source. Phospholipids were analyzed as the protonated molecule [M+H^+^] in positive ion mode; the mass spectrometer was set to scan Single ion Monitoring on m/z 594.3 (POVPC), 610.3 (PGPC), 782.5 (PAPC) and 828.5 (PEIPC). The results are expressed as ng/mg PAPCs.

### Statistical methods

The experiments were performed in triplicate and repeated for three times. Results were expressed as means ± SD. The statistical analysis was assessed by T-Student test for independent samples. In all analyses, a *p* value less than 0.05 (p<0.05) was considered a significant difference. Statistical analyses were performed by using SPSS for Windows version 16.0 (SPSS Inc., Chicago, IL, USA).

## Supporting Information

Table S1 **Fold change of mRNA levels in hMSCs treated for 48 hours with ox-PAPCs during adipogenic (** ***ADIPOQ*** **and** ***LEP*** **) and osteogenic (** ***SPP1*** **,** ***COLIA1*** **,** ***SPARC*** **) differentiation.**
(DOC)

Table S2 **Ox-PAPC values in patients (PTS) and controls (CNT).**
(DOC)

Table S3 **Fold change mRNA levels of pooled patients in hMSCs treated with OP vs. ND sera during adipogenic (** ***PPARγ2*** **,** ***ADIPOQ*** **and** ***LEP*** **) and osteogenic (** ***RUNX2*** **,** ***SPP1*** **,** ***COLIA1*** **,** ***SPARC*** **) differentiation.**
(DOC)
