# Supplementary material for: Role of Ox-PAPCs in the Differentiation of Mesenchymal Stem Cells (MSCs) and Runx2 and PPARγ2 Expression in MSCs-Like of Osteoporotic Patients
Source: PLoS One. 2011 Jun 3;6(6):e20363. doi: 10.1371/journal.pone.0020363 (PMC3108593; doi:10.1371/journal.pone.0020363)
Supplement: Table S1 — Fold change of mRNA levels in hMSCs treated for 48 hours with ox-PAPCs during adipogenic ( ADIPOQ and LEP ) and osteogenic ( SPP1 , COLIA1 , SPARC ) differentiation. (DOC) [file pone.0020363.s001.doc]

Table S1 **FOLD CHANGE mRNA LEVELS IN hMSCs TREATED WITH OX-PAPCS DURING ADIPOGENIC (ADIPOQ AND LEP) OR OSTEOGENIC (SPP1, COLIA1 AND SPARC) DIFFERENTIATION; *p<0.05**

| 48 treatment | Ox-PAPC 5 μg/ml | Ox-PAPC 10 μg/ml | Ox-PAPC 20 μg/ml |
| --- | --- | --- | --- |
| *AdipoQ* | 1.03 | 1.06 | 1.08 |
| *Lep* | 0.98 | 1.03 | 1.01 |
| *Spp1* | 1.02 | 0.98 | 1.01 |
| *Colia1* | 1.01 | 0.98 | 0.97 |
| *Sparc* | 0.97 | 1.01 | 0.98 |
