# Supplementary material for: Role of Ox-PAPCs in the Differentiation of Mesenchymal Stem Cells (MSCs) and Runx2 and PPARγ2 Expression in MSCs-Like of Osteoporotic Patients
Source: PLoS One. 2011 Jun 3;6(6):e20363. doi: 10.1371/journal.pone.0020363 (PMC3108593; doi:10.1371/journal.pone.0020363)
Supplement: Table S2 — Ox-PAPC values in patients (PTS) and controls (CNT). (DOC) [file pone.0020363.s002.doc]

**TABLE S2**.

| **PTS** | **POV/PAPC** | **PGPC/PAPC** | **PEIPC/PAPC** | **CNT** | **POV/PAPC** | **PGPC /PAPC** | **PEIPC/PAPC** |
| --- | --- | --- | --- | --- | --- | --- | --- |
| **1** | 1,62 | 0,59 | 3,50 | **1** | 1,08 | 0,54 | 3,25 |
| **2** | 1,49 | 0,66 | 0,39 | **2** | 1,15 | 0,54 | 3,44 |
| **3** | 1,35 | 0,49 | 0,61 | **3** | 1,21 | 0,53 | 2,79 |
| **4** | 1,52 | 0,60 | 3,50 | **4** | 1,35 | 0,41 | 3,84 |
| **5** | 1,26 | 0,62 | 1,79 | **5** | 1,23 | 0,49 | 2,66 |
| **6** | 1,67 | 0,57 | 3,65 | **6** | 1,20 | 0,58 | 2,68 |
| **7** | 1,65 | 0,49 | 3,63 | **7** | 1,38 | 0,51 | 2,58 |
| **8** | 1,08 | 0,60 | 3,60 | **8** | 1,68 | 0,49 | 3,06 |
| **9** | 1,29 | 0,61 | 3,61 | **9** | 1,21 | 0,52 | 2,36 |
| **10** | 1,42 | 0,58 | 3,65 | **10** | 1,19 | 0,51 | 3,39 |
| **11** | 1,17 | 0,65 | 3,60 | **11** | 1,18 | 0,53 | 2,34 |
| **12** | 1,26 | 0,62 | 3,61 | **12** | 1,18 | 0,54 | 3,66 |
| **13** | 1,32 | 0,60 | 4,10 | **13** | 0,87 | 0,51 | 2,58 |
| **14** | 1,19 | 0,58 | 5,87 | **14** | 1,24 | 0,55 | 2,64 |
| **15** | 1,36 | 0,61 | 3,64 | **15** | 1,17 | 0,53 | 2,18 |
| **16** | 1,25 | 0,61 | 3,54 | **16** | 1,19 | 0,52 | 2,27 |
| **17** | 1,26 | 0,61 | 3,51 | **17** | 1,12 | 0,52 | 3,58 |
| **18** | 1,40 | 0,63 | 3,55 | **18** | 1,18 | 0,53 | 2,74 |
| **19** | 1,20 | 0,62 | 6,60 | **19** | 1,12 | 0,54 | 2,98 |
| **20** | 1,40 | 0,59 | 3,52 | **20** | 1,22 | 0,51 | 3,65 |
| **21** | 1,30 | 0,59 | 3,52 | **21** | 1,23 | 0,54 | 2,89 |
| **22** | 1,31 | 0,62 | 3,53 | **22** | 1,21 | 0,55 | 3,35 |
| **23** | 1,24 | 0,61 | 3,58 | **23** | 1,20 | 0,51 | 2,82 |
| **24** | 1,35 | 0,63 | 3,61 | **24** | 1,22 | 0,53 | 3,86 |
| **25** | 1,35 | 0,62 | 3,55 | **25** | 1,10 | 0,55 | 3,00 |
| **26** | 1,38 | 0,60 | 3,52 |  |  |  |  |
| **27** | 1,32 | 0,62 | 3,52 |  |  |  |  |
| **28** | 1,42 | 0,60 | 3,50 |  |  |  |  |
| **29** | 1,35 | 0,62 | 3,55 |  |  |  |  |
| **30** | 1,30 | 0,61 | 3,56 |  |  |  |  |
| **31** | 1,33 | 0,63 | 3,56 |  |  |  |  |
| **32** | 1,33 | 0,61 | 3,50 |  |  |  |  |
| **33** | 1,30 | 0,60 | 3,53 |  |  |  |  |
| **34** | 1,38 | 0,62 | 3,58 |  |  |  |  |
