# Supplementary material for: Role of Ox-PAPCs in the Differentiation of Mesenchymal Stem Cells (MSCs) and Runx2 and PPARγ2 Expression in MSCs-Like of Osteoporotic Patients
Source: PLoS One. 2011 Jun 3;6(6):e20363. doi: 10.1371/journal.pone.0020363 (PMC3108593; doi:10.1371/journal.pone.0020363)
Supplement: Table S3 — Fold change mRNA levels of pooled patients in hMSCs treated with OP vs. ND sera during adipogenic ( PPARγ2 , ADIPOQ and LEP ) and osteogenic ( RUNX2 , SPP1 , COLIA1 , SPARC ) differentiation. (DOC) [file pone.0020363.s003.doc]

**TABLE S3.**

| 24h  OP sera treatment | **POOL A**  **(PTS 1, 3, 7, 14, 23, 29)** | **POOL B**  **(PTS 2, 11, 13, 18, 28, 34)** | **POOL C**  **(PTS 4, 9, 16, 25, 31, 33)** | **POOL D**  **(PTS 5, 12, 15, 20, 22, 26)** | **POOL E**  **(PTS 6, 10, 17, 24, 27, 30)** | **POOL F**  **(PTS 8, 19, 21, 32, 23, 29)** |
| --- | --- | --- | --- | --- | --- | --- |
| ***PPARγ2*** | 1.06 | 1.02 | 1.08 | 0.98 | 1.02 | 1.04 |
| ***AdipoQ*** | 1.02 | 1.03 | 0.98 | 0.97 | 1.04 | 1.02 |
| ***Lep*** | 1.04 | 1.02 | 0.98 | 1.02 | 0.97 | 1.05 |
| ***Runx2*** | 1.03 | 0.98 | 0.98 | 0.98 | 0.97 | 0.94 |
| ***Colia1*** | 0.98 | 0.98 | 1.02 | 1.03 | 0.89 | 0.92 |
| ***Sparc*** | 1.08 | 0.98 | 0.98 | 1.02 | 1.03 | 0.98 |
| ***Spp1*** | 1.15 | 0.98 | 1.06 | 1.05 | 0.98 | 1.1 |

| 48h  OP sera treatment | **POOL A**  **(PTS 1, 3, 7, 14, 23, 29)** | **POOL B**  **(PTS 2, 11, 13, 18, 28, 34)** | **POOL C**  **(PTS 4, 9, 16, 25, 31, 33)** | **POOL D**  **(PTS 5, 12, 15, 20, 22, 26)** | **POOL E**  **(PTS 6, 10, 17, 24, 27, 30)** | **POOL F**  **(PTS 8, 19, 21, 32, 23, 29)** |
| --- | --- | --- | --- | --- | --- | --- |
| ***PPARγ2*** | 1.2 | 1.18 | 0.98 | 1.02 | 1.2 | 1.1 |
| ***AdipoQ*** | 1.1 | 1.03 | 0.97 | 1.02 | 1.04 | 0.98 |
| ***Lep*** | 1.09 | 1.12 | 0.96 | 0.94 | 1.08 | 0.94 |
| ***Runx2*** | 0.95 | 0.94 | 0.92 | 0.91 | 0.88 | 0.92 |
| ***Colia1*** | 0.86 | 0.85 | 0.86 | 0.85 | 0.82 | 0.81 |
| ***Sparc*** | 0.84 | 0.78 | 0.85 | 0.77 | 0.81 | 0.76 |
| ***Spp1*** | 0.8 | 0.72 | 0.74 | 0.78 | 0.76 | 0.76 |

| 1week OP sera treatment | **POOL A**  **(PTS 1, 3, 7, 14, 23, 29)** | **POOL B**  **(PTS 2, 11, 13, 18, 28, 34)** | **POOL C**  **(PTS 4, 9, 16, 25, 31, 33)** | **POOL D**  **(PTS 5, 12, 15, 20, 22, 26)** | **POOL E**  **(PTS 6, 10, 17, 24, 27, 30)** | **POOL F**  **(PTS 8, 19, 21, 32, 23, 29)** |
| --- | --- | --- | --- | --- | --- | --- |
| ***PPARγ2*** | 1.28 | 1.34 | 1.35 | 1.24 | 1.37 | 1.25 |
| ***AdipoQ*** | 1.2 | 1.23 | 1.05 | 0.98 | 1.24 | 1.4 |
| ***Lep*** | 1.2 | 1.1 | 1.1 | 0.84 | 1.04 | 1.46 |
| ***Runx2*** | 0.94 | 0.87 | 0.95 | 0.97 | 0.96 | 0.85 |
| ***Colia1*** | 0.85 | 0.84 | 0.8 | 0.84 | 0.84 | 0.82 |
| ***Sparc*** | 0.75 | 0.8 | 0.76 | 0.78 | 0.83 | 0.78 |
| ***Spp1*** | 0.72 | 0.75 | 0.8 | 0.71 | 0.68 | 0.68 |
